# Correction: The oxygen-resistant [FeFe]-hydrogenase CbA5H harbors an unknown radical signal

**DOI:** 10.1039/d2sc90140d

**Published:** 2022-07-14

**Authors:** Melanie Heghmanns, Andreas Rutz, Yury Kutin, Vera Engelbrecht, Martin Winkler, Thomas Happe, Müge Kasanmascheff

**Affiliations:** TU Dortmund University, Department of Chemistry and Chemical Biology Otto-Hahn-Straße 6 44227 Dortmund Germany muege.kasanmascheff@tu-dortmund.de; Ruhr University Bochum, Faculty of Biology and Biotechnology, Photobiotechnology Universitätsstr. 150 44801 Bochum Germany thomas.happe@ruhr-uni-bochum.de; Technical University of Munich Campus Straubing for Biotechnology and Sustainability, Professorship for Electrobiotechnology Uferstrasse 53 94315 Straubing Germany

## Abstract

Correction for ‘The oxygen-resistant [FeFe]-hydrogenase CbA5H harbors an unknown radical signal’ by Melanie Heghmanns *et al.*, *Chem. Sci.*, 2022, **13**, 7289–7294, https://doi.org/10.1039/D2SC00385F.

The authors realized that incorrect references were cited following the sentence “In conjunction with the signal's significant width, the frequency dependence clearly indicates spin–spin interaction between the F-clusters.” The correct references are shown below as ref. 1 and 2.

Additionally ref. 36 and 37 were reversed in the reference list. The correct ref. 36 is shown below as ref. 3 and the correct ref. 37 is shown below as ref. 4.

The Royal Society of Chemistry apologises for these errors and any consequent inconvenience to authors and readers.

## Supplementary Material

## References

[cit1] Bencini A. and Gatteschi D., Electron Paramagnetic Resonance of Exchange Coupled Systems, Springer Berlin Heidelberg, 1990, vol. 53

[cit2] More C., Camensuli P., Dole F., Guigliarelli B., Asso M., Fournel A., Bertrand P. (1996). JBIC, J. Biol. Inorg. Chem..

[cit3] Esselborn J. (2013). et al.. Nat. Chem. Biol..

[cit4] Roessler M. M., Evans R. M., Davies R. A., Harmer J., Armstrong F. A. (2012). J. Am. Chem. Soc..

